# Reasons for Permanent Teeth Extractions and Related Factors among Adult Patients in the Eastern Province of Saudi Arabia

**DOI:** 10.1155/2021/5534455

**Published:** 2021-02-22

**Authors:** Abdullah Aljafar, Hassan Alibrahim, Ammar Alahmed, Ahmad AbuAli, Muhammad Nazir, Ahmed Alakel, Khalid Almas

**Affiliations:** Department of Preventive Dental Sciences, College of Dentistry, Imam Abdulrahman Bin Faisal University, Dammam, Saudi Arabia

## Abstract

**Objective:**

To evaluate the reasons for permanent teeth extractions and related factors among adult patients visiting dental clinics in the Eastern Province of Saudi Arabia.

**Materials and Methods:**

This retrospective cross-sectional study included data of patients who underwent teeth extractions. Data were collected from public and private dental clinics in different cities of the Eastern Province of Saudi Arabia (January–March 2020). The demographic information of patients and their reasons for teeth extractions were obtained from participating dentists.

**Results:**

The study included data of 696 patients with 55.9% of males and 44.1% of females. The mean number of teeth extractions in the sample was 1.86 ± 1.8, and it increased significantly with advancing age (*P* ≤ 0.001). Saudi (1.97 ± 1.98) versus non-Saudi patients (1.55 ± 1.11) (*P*=0.02) and patients in public practice (2.03 ± 1.95) versus patients in private practice (1.50 ± 1.38) (*P* ≤ 0.001) showed significantly higher teeth extractions. Dental caries was the most common reason for teeth extractions (49.1%), followed by remaining roots (18.5%), periodontal disease (18.4%), and impactions (7.2%). Most commonly extracted teeth included tooth # 30 (9.8%), followed by tooth #16 (9.6%), tooth # 1 (8.8%), tooth # 19 (8.3%), and tooth # 4 (8.3%). On the other hand, upper incisors were the least commonly extracted teeth.

**Conclusions:**

Dental caries, remaining roots, periodontal disease, and impactions were the most common reasons for teeth extractions in our sample of patients. The extractions increased significantly with increasing age. Saudis and patients in public clinics underwent significantly higher teeth extractions. Measures aimed at the prevention and treatment of oral conditions can help reduce teeth extractions and improve the quality of life of patients.

## 1. Introduction

Globally, total tooth loss affected 276 million people in 2015, and these estimates are expected to increase in the future due to population growth and aging [1]. Loss of permanent teeth can lead to masticatory dysfunction, pronunciation difficulty, malnutrition, limited choices of food, social isolation, and poor quality of life [2–4]. Tooth loss is a risk factor for cardiovascular disease and stroke, and there is a significant dose-response relationship between tooth loss and cardiovascular disease and stroke [5]. The literature also indicates that patients with tooth loss are 1.34 times more likely to develop dementia [2]. Recently, researchers confirmed a relationship between tooth loss susceptibility to all-cause mortality [6]. According to the Ministry of Health, 18,640 permanent teeth were extracted in 2019 in the Eastern Province of Saudi Arabia [7]. It was also reported that the numbers of extracted permanent teeth increased from the year 2011 to 2015 in the country [8].

Many recent studies conducted in adults around the world showed that caries was the leading cause of tooth extraction followed by periodontal disease [9–13]. A study conducted in Riyadh Saudi Arabia found that 50.2% of permanent teeth were extracted due to dental caries, and this was followed by orthodontic problems (18.2%), eruption problems (17.5%), and periodontal problems (8.2%) [14]. In Jizan, Saudi Arabia, dental caries was the most common reason for teeth extractions in younger patients whereas periodontal disease was the major reason for teeth extractions in older patients [15]. Dental caries accounted for 89.8% of teeth extracted in a study of 949 patients in Madinah [16]. A study evaluated reasons for teeth extractions in rural and urban populations of Al-Baha city. The authors showed that dental caries was the most frequent reason for teeth extraction in young patients whereas most teeth were extracted due to periodontal reasons in patients above 40 years of age [17].

Teeth extractions are very common in the Eastern Province of Saudi Arabia. However, the reasons for teeth extractions were not fully investigated. Therefore, the study aimed to evaluate the reasons for teeth extraction among adult populations in the Eastern Province of Saudi Arabia. The study will also explore the patterns of teeth extractions in private and public dental clinics. The study will inform policy makers to establish preventive programs to reduce the prevalence of teeth extractions. In addition, data may be used for the planning of oral health services and the dental workforce and revising dental curricula. This can also help decrease the cost of treatment and minimize the harmful effects of tooth loss.

## 2. Materials and Methods

This retrospective cross-sectional study included data of patients who underwent teeth extractions during the last one month. Patient data were obtained from oral surgeons and dentists practicing in different settings (governmental hospitals, governmental primary health care centers, private clinics, and teaching hospitals) in the Eastern Province of Saudi Arabia. The researchers visited these settings in person for data collection. A convenience sample was used for the selection of participating oral surgeons and dentists. The study included those oral surgeons and dentists who have been practicing in the province and provided their informed consent. After discussing the purpose and modalities of the study, a record form was handed to study participants. Researchers assured anonymity, privacy, and confidentiality of research data. The record form was printed in English language.

The information in the record form can be divided into two sections. The first section was filled by the participating dentists, which included the date of the extraction, patient's age, gender, tooth number, and the reasons for teeth extractions. The reasons for teeth extractions were assigned as the following groups: caries, remaining roots, periodontal diseases, orthodontic, prosthodontic, impaction, trauma, cosmetic/esthetic, cysts, and others. The second section was filled by the interns, and it included the sector (governmental, private, or educational) and the location of dental practice.

The researchers collected data from participating dentists on weekly basis during a period of three months (Jan–March 2020). Some oral surgeon and dentists returned the completed record form during the first visit of researchers. Those participants who were unable to provided patients data were visited again after one-two weeks. A maximum of three visits were made to those dentists who were busy and were unable to provide data in the first and second visits. The study was conducted in accordance with ethical principles of the Declaration of Helsinki.

Data were entered in MS Excel (2010) and then transferred to IBM SPSS for Windows version 22.0 (IBM Corp., Armonk, N.Y., USA) for statistical analysis. Descriptive statistics included frequencies, percentages, means, and standard deviations. The Pearson chi-square test was used to compare differences in the proportions of reasons for teeth extractions between males versus females, Saudi patients versus non-Saudi patients, and patients visiting the public sector versus the private sector. A Kolmogorov–Smirnov test was performed to confirm the normality of quantitative variable, and accordingly, a *t*-test and one-way ANOVA test were used in the study. An independent *t*-test was performed to compare the mean number of extracted teeth in male and female patients, Saudi and non-Saudi patients, and patients in public and private sectors. One-way ANOVA was used to compare mean differences in the number of teeth extractions in different categories of age. A *P* value of less than 0.05 was used for statistical significance.

## 3. Results

The study included data analysis of 696 patients with 64.5% in the age range of 25–54 years. There were 55.9% of males and 44.1% of females with 73.7% of Saudi patients in the study. More patients (67.4%) were from public practice than private practice (32.6%) (Table 1).

The mean number of teeth extractions in the sample was 1.86 ± 1.8. The study found a statistically significant difference in the mean number of extracted teeth in different age groups (*P* ≤ 0.001), and the mean number of teeth extractions increased with advancing age. A significantly higher mean number of extracted teeth was found in Saudi (1.97 ± 1.98) versus non-Saudi patients (1.55 ± 1.11) (*P*=0.02) and patients in public (2.03 ± 1.95) versus patients in private practice (1.50 ± 1.38) (*P* ≤ 0.001). Males had a greater mean number of extracted teeth than females, but the difference was not statistically significant (*P*=0.517) (Table 2).

**Figure 1:**
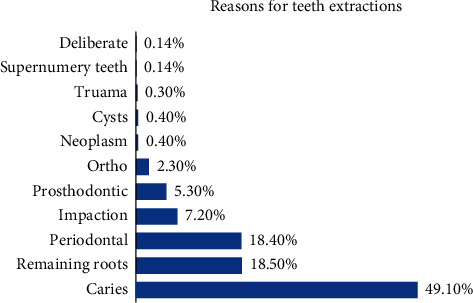
Distribution of reasons for teeth extractions in patients.

Dental caries was the most common reason for teeth extractions (49.1%), followed by remaining roots (18.5%), periodontal disease (18.4%), and impactions (7.2%) (Figure 1).

Table 3 shows the relationships of reasons for teeth extractions with gender, nationality, and type of dental practice. Higher proportions of males compared with females had teeth extracted due to caries, periodontal disease, remaining roots, impactions, and prosthodontics; however, differences were not statistically significant. A significantly higher proportion of Saudi (68.4%) than non-Saudi patients (31.6%) had tooth extracted due to caries (*P*=0.002). Overall, more Saudi versus non-Saudi patients had teeth extractions due to periodontal disease, remaining roots, impactions, and prosthodontics, but differences were not statistically significant (*P* > 0.05). The patients in public clinics had significantly greater extractions due to caries than those in private practice (*P*=0.003). On the other hand, more orthodontic extractions were performed in private than public practice, and the difference was statistically significant (*P*=0.041).

**Figure 2:**
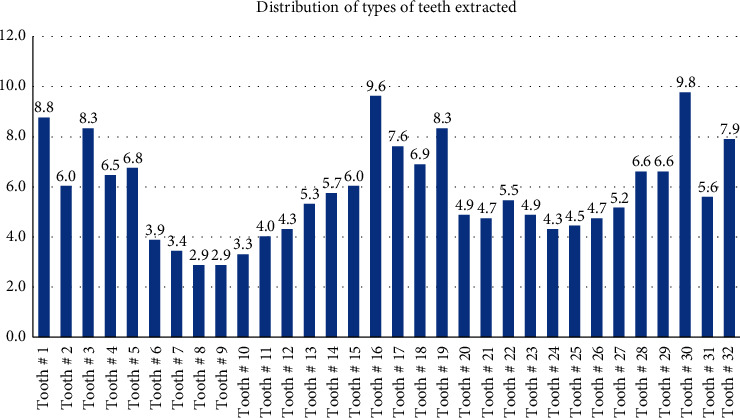
Distribution of type of teeth extracted among patients.

Figure 2 presents the distribution of the types of teeth extracted among patients. Most commonly extracted teeth included tooth # 30 (9.8%), followed by tooth #16 (9.6%), tooth # 1 (8.8%), tooth # 19 (8.3%), and tooth # 4 (8.3%). On the other hand, upper incisors were the least commonly extracted teeth in the study.

**Figure 3:**
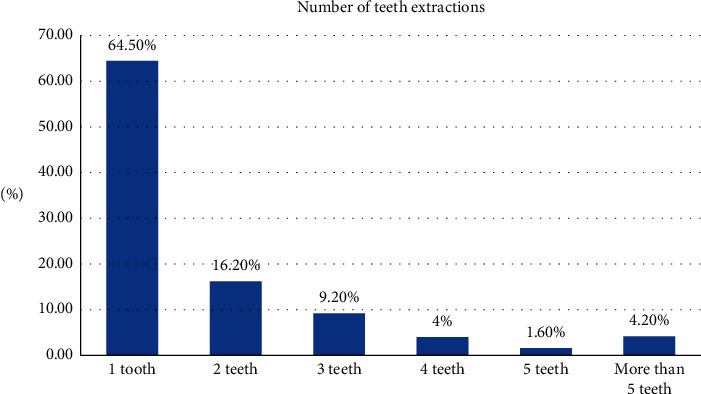
Distribution of the number of teeth extractions in patients.

Figure 3 shows the number of teeth extractions in patients. Two-thirds of patients (64.5%) had one tooth extracted, 16.2% had two teeth extracted, and 9.2% had three teeth extracted. The extractions of more than 5 teeth were reported in 4.2% of patients.

## 4. Discussion

Dental caries and periodontal disease were the most common reasons for teeth extractions in the present study. Similar findings were reported in previous studies by Lesolang et al. [10] in South Africa, Byahatti and Ingafou [11] in Libya, Al-Shammari et al. [12] in Kuwait, Anand and Kuriakose [18] in India, Zafar et al. [19] in Pakistan, Taiwo et al. [9] in Nigeria, Lee et al. [20] in Taiwan, Morita et al. [13] in Japan, Angelillo et al. [21] in Italy, and Ong et al. [22] in Singapore. In Saudi Arabia, Asmat et al. also found that caries was the most frequent reason for teeth extractions followed by periodontal disease [23]. Gossadi et al. indicated caries as the main reason for teeth extractions in 20–29 year-old patients, and periodontal diseases were the most common reasons for teeth extraction in 40–59-year-old patients in Gizan, Saudi Arabia [15]. However, Alaboudi et al. reported that caries was responsible for 89.8% of teeth extractions in Madinah, Saudi Arabia [16].

The high prevalence of dental caries and periodontitis globally and locally explains the reasons for these findings in the international and local literature. Globally, dental caries and periodontal disease are widely distributed in different populations. According to the Global Burden of Disease Study (2017), 3.47 billion people suffered from oral disorders and dental caries in permanent teeth was the most common health condition affecting 2.3 billion people around the globe [24]. Similarly, severe periodontitis was the sixth most common condition affecting 10.8% of the world population in 2010 [25]. Locally, a study reported caries prevalence of 89.2% in adult patients in the Al-Ahsa region of Saudi Arabia [26]. Similarly, a high prevalence of periodontitis (36.8%) was observed in adult patients in Abha, Saudi Arabia [27]. It should be noted that the provision of dental care for caries or periodontal disease can prevent tooth loss. However, the prevention of tooth loss may result in a simultaneous increase in the prevalence and incidence of caries and periodontitis [28]. It is known that the direct treatment cost of oral conditions was US$298 billion in 2010 which represented 4.6% of healthcare expenditure worldwide [29]. Therefore, efforts should be focused on preventive measures to prevent caries, periodontitis, and other oral conditions.

Dental caries is a disease of the adults, and the incidence rate of dental caries in adults is as great as in adolescents and children [30]. However, dental caries being a chronic and cumulative disease increases with age [31]. The distribution of periodontal disease also varies in different life spans of an individual. Globally, the prevalence of periodontitis (periodontal pocket of 4-5 mm) is 9.1% in adolescents, 27.7% in adults, and 30.6% in older persons [32]. It is known that the severe periodontitis increases with advancing age, reaches its peak at 40 years of life, and stabilizes in old age [33]. Dental caries and periodontal diseases are the main causes of teeth extractions, and the distributions of these conditions increase with age. This explains the reasons for greater teeth extractions with advancing age in the present study.

A previous study in Saudi Arabia showed that Saudi patients demonstrated significantly higher gingivitis than non-Saudi patients [27]. In the present study, Saudi patients underwent significantly higher teeth extraction compared with non-Saudi populations. Poor oral hygiene, increased consumption of fermentable carbohydrates, low routine dental attendance, low awareness about oral health programs, and tobacco consumption are common among Saudi populations, and these factors increase their predisposition to oral diseases which can eventually lead to tooth mortality [27, 34, 35].

Dental practitioners in private dental clinics charge a fee for the delivery of oral care from their patients or health insurance companies. They are more likely to provide more conservative, endodontic, periodontal, and other dental treatments to achieve patient satisfaction and greater earning/profits for their clinics. On the contrary, dentists in public clinics are paid by the government for the services provided to patients and may be less interested in providing conservative and time consuming dental treatment. In addition, dentists may be unable to provide treatment to a large number of patients visiting public dental clinics because dental care is free in Saudi Arabia [36]. Moreover, patients attending public dental clinics may not be aware of treatments available to save the tooth and, hence, may agree to tooth extraction rather than availing other treatment options. These may account for a significantly greater number of teeth extractions in public than in private clinics in the present study.

Mandibular and maxillary molars were the most frequently extracted teeth in the present study. Similar findings were observed in previous studies [9, 14, 19, 20]. The reasons for increased extractions of molars in both dentitions can be explained by their morphology, the timing of eruption, and position in the oral cavity. The presence of deep pits and fissures increases the susceptibility of molars to the accumulation of food debris and retention of plaque which increases the risk of caries. First, permanent molars erupt earlier in the oral cavity which predisposes them to acid attack more than other teeth. In addition, caries in the first permanent molars can lead to caries in the second molars, premolars, and incisors [37]. The researchers suggest an association between caries and periodontal disease and excessive intake of fermentable carbohydrates can account for the development of caries and inflammation of periodontal tissues [38]. Additionally, the position of molars in the oral cavity can affect one's ability to maintain proper oral hygiene and plaque control resulting in caries and tooth loss. On the other hand, anterior teeth were least frequently extracted teeth in the present study. The morphology of anterior teeth and cleansing and buffering actions due to greater availability of saliva from submandibular and sublingual salivary glands may explain the reasons for their reduced extractions [39].

The study added valuable information from the Eastern Province of Saudi Arabia to the existing knowledge base on the reasons for permanent teeth extractions. However, there are certain limitations to the study. The findings of the study should not be generalized to populations in other regions in Saudi Arabia. Moreover, self-reported data also are subjected to biases in the study.

## 5. Conclusions

The study found that dental caries was the most common reason for teeth extractions, followed by periodontal disease. Maxillary and mandibular molars were the most frequently extracted teeth. Saudis and patients in public clinics underwent greater extractions compared with non-Saudis and patients in private practice. The number of extractions enhanced with an increase in the age of patients. Oral health-care systems should focus on the effective prevention and treatment of oral conditions particularly caries and periodontal disease to reduce the burden of teeth extractions and improve the quality of life of patients.

## Figures and Tables

**Table 1:** Demographic information of patients (*N* = 696).

Variables	Patients, *N* (%)
*Age*	
15–24	113 (16.2)
25–34	198 (28.4)
35–44	125 (18.0)
45–54	126 (18.1)
55–64	87 (12.5)
65 and above	47 (6.8)

*Gender*	
Male	389 (55.9)
Female	307 (44.1)

*Nationality*	
Saudi	513 (73.7)
Non-Saudi	183 (26.3)

*Type of practice*	
Public	469 (67.4)
Private	227 (32.6)

**Table 2:** Relationship between demographic factors and teeth extractions.

Variables	Mean number of extracted teeth Mean ± SD	*P* value
*Age*		
15–24	1.28 ± 0.55	<0.001
25–34	1.45 ± 1.00	
35–44	2.06 ± 2.44	
45–54	2.06 ± 1.79	
55–64	2.30 ± 2.17	
65 and above	3.04 ± 2.60	

*Gender*		
Male	1.96 ± 2.09	0.517
Female	1.73 ± 1.35	

*Nationality*		
Saudi	1.97 ± 1.98	0.02
Non-Saudi	1.55 ± 1.11	

*Type of practice*		
Public	2.03 ± 1.95	<0.001
Private	1.50 ± 1.38	

**Table 3:** Distribution of reasons for teeth extractions among patients by gender, nationality, and type of dental practice.

	Male, *N* (%)	Female, *N* (%)	*P* value	Saudi, *N* (%)	Non-Saudi, *N* (%)	*P* value	Public, *N* (%)	Private, *N* (%)	*P* value
Caries	198 (57.9)	144 (42.1)	0.295	234 (68.4)	108 (31.6)	0.002	249 (72.8)	93 (27.2)	0.003
Periodontal	68 (53.1)	60 (46.9)	0.485	99 (77.3)	29 (22.7)	0.301	81 (63.3)	47 (36.7)	0.125
Remaining roots	65 (50.4)	64 (49.6)	0.163	100 (77.5)	29 (22.5)	0.276	76 (58.9)	53 (41.1)	0.023
Impactions	32 (64)	18 (36)	0.231	37 (74)	13 (26)	0.752	35 (70)	15 (30)	0.682
Prosthodontic	22(59.5)	15 (40.5)	0.653	27 (73)	10 (27)	0.917	29 (78.4)	8 (21.6)	0.143
Orthodontics	8 (50)	8 (50)	0.631	16 (100)	0	0.016 Fisher	7 (43.8)	9 (56.3)	0.041

## Data Availability

The SPSS data file of this study is available from the corresponding author upon request.
